# Transforming growth factor-β receptors: versatile mechanisms of ligand activation

**DOI:** 10.1038/s41401-024-01235-6

**Published:** 2024-02-13

**Authors:** Zheng-Jie Chia, Ying-nan Cao, Peter J. Little, Danielle Kamato

**Affiliations:** 1 School of Pharmacy, The University of Queensland, Brisbane, QLD 4102 Australia; 2 Discovery Biology, School of Environment and Science, Griffith University, Brisbane, QLD 4111 Australia; 3 Griffith Institute for Drug Discovery, Griffith University, Brisbane, QLD 4111 Australia; 4 Department of Pharmacy, Guangzhou Xinhua University, Guangzhou, 510520 China

**Keywords:** TGFBR, receptor Smads, thrombospondins, matrix metalloproteinases, integrins, transactivation

## Abstract

Transforming growth factor-β (TGF-β) signaling is initiated by activation of transmembrane TGF-β receptors (TGFBR), which deploys Smad2/3 transcription factors to control cellular responses. Failure or dysregulation in the TGF-β signaling pathways leads to pathological conditions. TGF-β signaling is regulated at different levels along the pathways and begins with the liberation of TGF-β ligand from its latent form. The mechanisms of TGFBR activation display selectivity to cell types, agonists, and TGF-β isoforms, enabling precise control of TGF-β signals. In addition, the cell surface compartments used to release active TGF-β are surprisingly vibrant, using thrombospondins, integrins, matrix metalloproteinases and reactive oxygen species. The scope of TGFBR activation is further unfolded with the discovery of TGFBR activation initiated by other signaling pathways. The unique combination of mechanisms works in series to trigger TGFBR activation, which can be explored as therapeutic targets. This comprehensive review provides valuable insights into the diverse mechanisms underpinning TGFBR activation, shedding light on potential avenues for therapeutic exploration.

## Introduction

Transforming growth factor-β (TGF-β) is a ubiquitously expressed cytokine that plays a vital role in regulating a variety of cellular events ranging from organogenesis, differentiation, cell growth to hemostasis, extracellular matrix production, and immune responses [1, 2]. Regulation of TGF-β signals is required to maintain the physiological status of cells [3]. Uncontrolled TGF-β signaling pathways have been linked to fibrosis [4, 5], cancers [4, 5], atherosclerosis [6], systemic sclerosis [7], early-stage myelodysplastic syndromes [8] and inflammation in bowel tissue and the central nervous system [9].

**Fig. 1:**
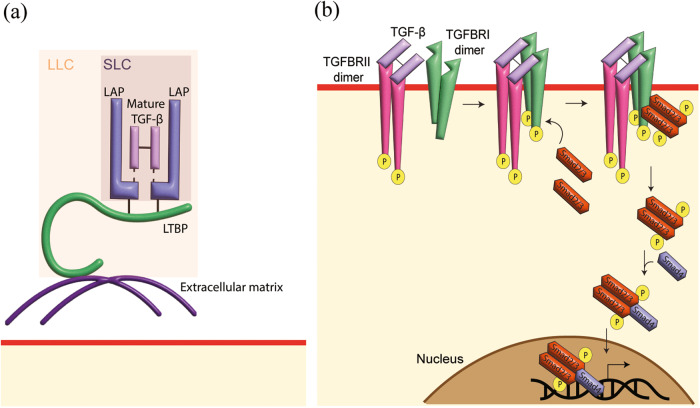
The TGF-β complex and TGFBR signaling pathways. **a** Transforming growth factor (TGF)-β is synthesized as pro-TGF-β peptide before being cleaved to form latency associated peptide (LAP) and mature TGF-β ligand. The mature TGF-β is non-covalently associated with LAP, forming small latent complex (SLC). SLC is then linked to latent TGF-β binding protein (LTBP) to form large latency complex (LLC) before being secreted to the extracellular domain and deposited in the extracellular matrix. **b** Active TGF-β will first bind to transforming growth factor-β receptor II (TGFBRII) dimer before forming a complex with transforming growth factor-β receptor I (TGFBRI) dimer. Activated TGFBRI recruits Smad2/3 and phosphorylates the carboxyl-terminal of Smad2/3. Phosphorylated Smad2/3 will associate with Smad4 and other co-factors before forming a transcription factor complex to regulate gene expression.

TGF-β occurs as five isoforms [10], with TGF-β1, TGF-β2 and TGF-β3 expressed in most mammals and TGF-β4 and TGF-β5 found only in chicken and frogs, respectively [11, 12]. This review will focus on mammalian TGF-β isoforms. TGF-β is present in the extracellular matrix as inactive forms [13]. Mature TGF-β non-covalently interacts with latency associated peptide (LAP), forming the small latent complex (SLC) [14]. The SLC then undergoes the addition of latent TGF-β binding proteins (LTBP-1, -3, and -4) via disulfide bonds to form a large latent complex (LLCs). LTBPs have preferences for different TGF-β isoforms. TGF-β1 has been found to interact with LTBP-1, -3, -4, whereas TGF-β2 and TGF-β3 only form complexes with LTBP-1 and -3 [13, 15]. LTBPs deposit TGF-β in the extracellular matrix and facilitate the release of TGF-β [13, 16] (Fig. 1a). Several cell surface mechanisms, such as integrins, matrix metalloproteinases, and thrombospondins, are engaged directly or in combination to release the active TGF-β from latent forms to activate transforming growth factor-β receptors (TGFBRs) [17–19].

To elicit a cellular response, TGF-β signals through transmembrane TGFBRs, recognized for their serine/threonine kinase activities [20]. Once released, the active TGF-β must bind to the TGFBRs to elicit downstream signaling. There are two types of TGFBRs—TGFBRI and TGFBRII, which are structurally and functionally distinct [21, 22]. The ligand-bound TGFBRII dimer will recruit the TGFBRI dimer and phosphorylate the serine and threonine residues in the GS domain of TGFBRI to activate TGFBRI [1, 23]. Upon the activation of TGFBRI, unphosphorylated Smad2/3 will be delivered to the phosphorylated TGFBRI to facilitate the phosphorylation of the carboxyl-terminal of Smad2 (Serine 465/467) and Smad3 (Serine 423/435) [24–27]. Phosphorylated Smad2 or Smad3 will dissociate from activated TGFBRs and form a complex with Smad4 and other co-factors such as Fos and Jun before traveling to the nucleus to modulate gene expressions [28, 29] (Fig. 1b).

The release of active TGF-β can occur via thrombospondin-1, F-spondin, neuropilin-1, integrins, matrix metalloproteinases and reactive oxygen species-dependent pathways. Activation of other cell surface receptors, which employ these same signaling intermediates, can lead to the release of the active TGF-β and activation of TGFBR signaling in a process known as transactivation-dependent signaling [30]. The recruitment of different mechanisms to activate TGFBR is highly selective to cell types and agonists, enabling the development of targeted therapy to inhibit TGF-β signals. This review will present the transactivation-dependent and -independent mechanisms of TGFBR activation that lead to the release of the active TGF-β from its complex and provide insights into the implications for potential targets to inhibit TGFBR activation.

## Release of active TGF-β via the actions of KRFK motif-containing proteins

**Fig. 2:**
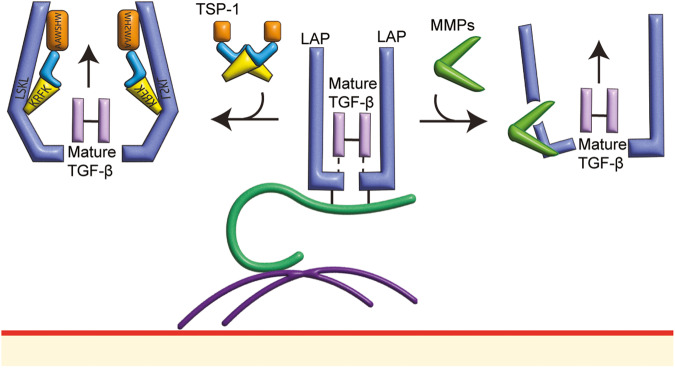
Liberation of mature TGF-β by TSP-1 and MMPs. Left side: Thrombospondin-1 (TSP-1) is a protein found in the extracellular matrix. The AAWSHW domain in TSP-1 can recognize the latency associated peptide (LAP) portion of the transforming growth factor (TGF)-β complex and deposit TSP-1 to LAP, which will then disrupt the structure of LAP with its KRFK motif. The modified LAP loses the ability to maintain the latency of mature TGF-β, therefore releasing the mature TGF-β for transforming growth factor-β receptor (TGFBR) activation. Right side: Matrix metalloproteinases (MMPs) are enzymes that hydrolyze peptides into smaller sections. The LAP is susceptible to MMP proteolysis, thereby disturbing the structural integrity of LAP, enabling the release of mature TGF-β for TGFBR activation.

Thrombospondins (TSPs) are extracellular proteins with multifactorial roles in controlling cell activities by interacting with extracellular matrix and growth factors, including the release of active TGF-β [31–33]. To release active TGF-β from SLC, the AAWSHW domain on TSP-1 will first bind to the LAP, directing the KRFK motif on TSP-1 to the LSKL segment on LAP. This disrupts the interaction of mature TGF-β with LAP, allowing the liberation of active TGF-β [17, 34]. Administrating LSKL or AAWSHW peptides inhibits TGFBR activation and Smad2 phosphorylation in cardiac tissues [35] and glomerulus [34] in rats, demonstrating the critical role of the TSP-1 domains in the liberation of TGF-β (Fig. 2). TSP-2 shares approximately 60% similarity in peptide sequence to TSP-1. However, TSP-2 does not have the KRFK sequence required to release active TGF-β. When incubating TSP-2 and TSP-1 with SLC, the release of active TGF-β induced by TSP-1 is abolished [36]. In the glomerulus tissues of rats with nephritis, TSP-1 expression is enhanced, correlating with increased active TGF-β and Smad2 phosphorylation found in the tissues. The increase in TGF-β and Smad2 phosphorylation levels are drastically reduced when the TSP-2 gene is overexpressed, demonstrating that TSP-2 is an antagonist of TSP-1, regulating the TSP-1 mediated release of active TGF-β [37]. F-spondin is an extracellular matrix protein regulating cell adhesion that also carries the KRFK motif [38]. Treating knee joint explant obtained from osteoarthritis patients with F-spondin induces the release of active TGF-β without changing the level of total TGF-β, showing that KRFK-containing proteins can facilitate the liberation of active TGF-β from the latent TGF-β reservoir on the cell surface [39]. In the lung tissue of TSP-1^–/–^ mice [40] and human fibroblasts with TSP-1 knocked down [41], Smad2/3 is still phosphorylated, suggesting that different tissues employ different pathways to activate TGFBR.

A motif similar to KRFK, RKFK, is found on a transmembrane glycoprotein, neuropilin-1 [42]. The extracellular domain of neuropilin-1 facilitates the binding of growth factors to their receptors and carries the RKFK motif that can effectively release active TGF-β1 from SLC to the same extent as KRFK peptide (as in TSP-1) [42, 43]. In breast [44] and lung cancer cells [45], activation of TGFBR triggered by overexpression of neuropilin-1 leads to increased Smad2/3 activation, which is inhibited when neuropilin-1 is silenced. Neuropilin-1 also promotes TGF-β signaling by facilitating the complexation of TGFBR and the internalization of activated TGFBR for signal transduction [44, 46]. Examples from TSP-1, F-spondin and neuropilin-1 suggested that screening of proteins containing KRFK or RKFK motif in the extracellular domain can be useful to explore unknown mechanisms involved in TGFBR activation.

## Proteolytic release of active TGF-β by matrix metalloproteinases

Matrix metalloproteinases (MMPs) are a group of proteases that degrade the extracellular matrix, and their activities and functions are inhibited by tissue inhibitors of matrix metalloproteinases (TIMPs) [47–49]. Most MMPs are secreted from the cells, except for membrane-type MMPs, which are attached to the plasma membrane [50]. MMPs are secreted in an inactive pro-peptide form, which requires activation to act as endopeptidases to hydrolyze the peptide bond in the substrates [50, 51]. The SLC is one of the substrates targeted by MMPs.

MMP-2 induces the release of active TGF-β and enhances Smad2/3 phosphorylation in the rat aortic rings [19]. A similar result is observed in rat vascular smooth muscle cells, where MMP-2-mediated TGF-β release is completely inhibited by TIMP-2 and GM6001 (a universal MMP antagonist) [19]. MMP-9, another member of the gelatinase family, also promotes the liberation of active TGF-β. In human breast cancer cells, upregulation of MMP-9 increases the release of active TGF-β from SLC, resulting in enhanced Smad2 phosphorylation, which correlates with cancer cell proliferation [52]. Through catalyzing the hydrolysis of peptide bonds in SLC, MMPs facilitate the release of active TGF-β from SLC, ready to engage with TGFBRs to induce TGFBR activation (Fig. 2).

CD44 is a transmembrane glycoprotein expressed in many cancer cells, regulating cell and cytokine interactions [53, 54]. In human mammary cancer cells and mouse fetal myoblast coculture, the lack of expression of CD44 correlates with a reduction of TGFBR activation [55]. The activation of TGFBR is rescued when both CD44 and MMP-9/MMP-2 are co-expressed but not MMP-9 or MMP-2 alone [55]. These results demonstrate that MMP-2 and MMP-9 are directed by CD44 to the SLC complex on the cell surface to hydrolyze LAP to release active TGF-β.

MMPs and TIMPs regulate TGF-β signaling, and the failure of this regulation has been associated with glaucoma [56], arthritis [57], systemic sclerosis [58] and cancers [59]. TGF-β in multiple cell types upregulates the expression of TIMPs to antagonize the effect of MMPs. In chondrocytes [48, 60], human airway fibroblast [61], human breast cancer cells [59], pancreatic stellate cells [62], human epithelial cells [63] and human gingival fibroblast [64], treatment with TGF-β increases the synthesis of TIMP-1 or TIMP-3, implying that upon TGFBR activation, TIMPs synthesis is upregulated to counteract the action of MMPs. MMPs have been linked to metastasis as the breaking down of extracellular matrix enables the migration of cancer cells and cell adhesion, and it was thought that TIMPs could reverse the pro-metastatic effects of MMPs [65, 66]. However, the levels of TIMPs correlate with breast cancer invasiveness [59], and TIMPs are responsible for the proliferative effects of TGF-β in cancer cells. TGF-β treatment of hepatocellular carcinoma cells had no effect on cell proliferation; however, when the cells were exposed to media from TGF-β-treated hepatic stellate cells, an increase in cell proliferation and invasion was observed [67]. The removal of TIMP-1 by immunoprecipitation abolishes hepatocellular carcinoma cell proliferation and migration, showing that TIMP-1 relays pro-metastatic signals of TGF-β between different cells [67]. The same mechanism is also seen in the microenvironment of lung cancer in which invasion and proliferation of lung adenocarcinoma cells is induced by TIMP-1 secretion from TGF-β-treated tumor-associated fibroblast [68]. In pancreatic cancer, TGF-β secreted by macrophages can enhance the TIMP-1 secretion in pancreatic stellate cells, which increases the cell growth of pancreatic intraepithelial neoplasia and is associated with a more invasive phenotype [62]. TIMP-1 also plays a role in the profibrotic effect of TGF-β by inhibiting the breakdown of the extracellular matrix induced by MMPs to cause fibrosis. In the colon of rat with colitis, the levels of TGF-β and Smad2/3 phosphorylation increases with disease progression [69]. TIMP-1 activity increases with colitis progression, correlating with the thickening of the colon tissue and collagen deposition, in contrast the expressions of MMP-1 and MMP-3 are gradually reduced [69]. In mice with overexpression of TGF-β, the level of collagen in the left ventricular tissue and the protein expressions of TIMP-1,2,4 are enhanced, and the levels of active MMPs responsible for collagen catabolism are inhibited, suggesting the link between TGF-β/TIMPs and myocardial fibrosis [70]. While TIMPs negatively regulate the activities of MMPs, their roles in controlling TGF-β signaling in tumorigenesis and fibrosis might be synergistic, whereby MMPs initiate the release of active TGF-β from SLC, and the effects of TGF-β signaling in forming cancers and fibrosis are propagated with TIMPs.

## Release of active TGF-β by RGD motif-recognizing integrins

Integrins are transmembrane proteins consisting of α and β subunits [71]. The extracellular domain of α and β subunits are associated with ligand binding and extracellular matrix adhesion, whereas the intracellular domain of the α subunit acts as an anchor point, and the cytoplasmic domain of the β subunit connects with the cytoskeleton to enable conformational changes [71, 72]. Integrins with an arginine-glycine-aspartic acid motif (RGD motif) recognizing domain such as αvβ3, αvβ5, αvβ6 and αvβ8 can mediate the liberation of active TGF-β [73, 74]. LAPs of TGF-β1 (244-RGD) [74] and TGF-β3 (261-RGD) [75] have RGD motif that bind the α and β subunits. For the release of active TGF-β, integrins bind to the LAP of the TGF-β complex and release the TGF-β following cytoskeletal rearrangement [76] or the recruitment of extracellular proteins to hydrolyze the structure of LAP [77]. The LAP of TGF-β2 does not have an RGD motif. Instead, TGF-β2 has an SGD motif. Therefore, the release of active TGF-β2 cannot be mediated by RGD motif-recognizing integrins [75].

**Fig. 3:**
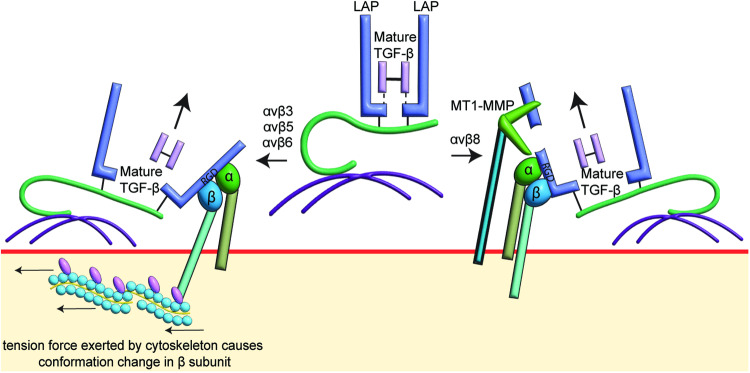
The liberation of mature TGF-β by RGD motif-recognizing integrins (αvβ3, αvβ5, αvβ6, αvβ8). RGD motif-recognizing integrins can identify the RGD domain on latency associated peptide (LAP). Left side: αvβ3, αvβ5 and αvβ6 upon identify the RGD domain on LAP, secure the LAP for tension force exerted by cytoskeleton, which physically disrupts the architecture of LAP, allowing the release of mature transforming growth factor (TGF)-β, which is ready to bind to transforming growth factor-β receptor (TGFBR). Right side: αvβ8 does not require cytoskeleton for the release of mature TGF-β. Upon αvβ8 secures the LAP, membrane type-1 matrix metalloproteinase (MT1-MMP) is used to cleave LAP, enabling mature TGF-β to escape from the LAP for the activation of TGFBR.

In liver fibroblasts [18] and SW480 colon adenocarcinoma cells [78], overexpressing the integrin β6 subunit increases the release of TGF-β. Treating β6-overexpressing SW480 colon adenocarcinoma cells with cytochalasin D to inhibit the cytoskeleton polymerization prevents the activation of TGFBR to the same extent as treating the cell with anti-αvβ6, showing that cytoskeleton rearrangement is required for the activation of TGFBR via αvβ6 (Fig. 3) [78].

Targeting of αvβ6-mediated TGFBR activation with broad-spectrum MMP inhibitors had no effect on TGFBR activation, showing that αvβ6 mediated TGFBR activation occurs independently of MMPs [77]. For αvβ6 to facilitate the release of active TGF-β, LTBP is required. In liver fibroblasts engineered to express the β6 subunit of integrins, inhibiting the association of LAP and LTBP-1 by mutating the LTBP-1 binding sites on LAP suppresses the release of active TGF-β dramatically [18]. Modifications of the N-terminal of LTBP-1 show that the 402–449 hinge region is essential for LTBP-1 to mediate the release of active TGF-β [18]. The 402–449 hinge region is unique to LTBP-1 but not in other LTBPs. In liver fibroblasts overexpressing LTBP-3, no αvβ6-mediated release of active TGF-β is observed. However, when the hinge region in LTBP-3 is replaced with the LTBP-1 hinge region sequence, LTBP-3 can facilitate the release of active TGF-β, demonstrating the αvβ6-mediated release of active TGF-β is specific to LTBP-1 bound TGF-β [18]. In TGF-β reporter cells, the amount of active TGF-β released by αvβ6 can only be detected when the TGF-β reporter cells are co-cultured with the αvβ6-mediated TGF-β releasing cells but not treating TGF-β reporter cells with media collected from activated cells, suggesting that αvβ6 is used by cells to initiate paracrine TGF-β signaling [78]. Capan-2 human pancreatic adenocarcinoma cells do not release active TGF-β. However, co-culturing of Capan-2 with F99 fibroblasts enhances the release of active TGF-β, which is inhibited by an αvβ6 antibody, 3G9 [79]. These studies demonstrate that αvβ6-mediated release of active TGF-β is a specific mechanism for localized TGF-β signaling.

αvβ6 in different cells prefer to activate the release of different TGF-β isoforms. In HT-1080 fibrosarcoma cells [80] and SW480 colon adenocarcinoma cells [80], αvβ6 mediates the release of TGF-β1 and TGF-β3. However, in liver fibroblast cells [78], αvβ6 only facilitates the release of TGF-β1. Similar to αvβ6, the αvβ5 and αvβ3 require rearrangement of cytoskeleton to mediate the release of active TGF-β. The active TGF-β can only be detected when cells are co-cultured with reporting cells [81–83], indicating αvβ6, αvβ5 and αvβ3 are responsible for local TGF-β responses.

In human airway epithelial cells [84] and human fetal astrocytes [85], the expression of the β8 subunit is remarkably higher than β6, and inhibition of the β8 subunit greatly reduces the release of active TGF-β. In αvβ6-mediated TGFBR activation, the cytoskeleton is required to achieve conformation changes in the SLC to trigger the release of active TGF-β. However, the cytoplasmic region of αvβ8 is not required to activate TGFBR in SW480 adenocarcinoma cells and HT1080 fibrosarcoma cells [77]. This shows a different mechanism, which is independent of the cytoskeleton. Instead, to facilitate the release of active TGF-β, αvβ8 works with MMPs to cleave the LAP in SLC to allow the release of active TGF-β (Fig. 3). In SW480 adenocarcinoma cells [77] and human fetal astrocytes [85], GM6001 inhibits the release of active TGF-β. Inactivating membrane type-1 matrix metalloproteinases (MT1-MMP) drastically reduced the release of active TGF-β in αvβ8 overexpressing H1264 lung cancer cells, indicating that MT1-MMP and αvβ8 work together to activate TGFBR [77]. In αvβ6-mediated liberation of active TGF-β, active TGF-β can only be detected when TGF-β-releasing cells are cultured with reporting cells. However, in αvβ8-mediated liberation of active TGF-β, TGF-β is readily detected by treating the reporting cells with human astrocytes [85] and fibrosarcoma [77], showing that αvβ8 is responsible for the activation of TGFBR in a broader area.

The choice of RGD motif-recognizing integrins for TGFBR activation depends on the cell type [86]. Incubating human fetal tracheal epithelial cells with anti-β3, anti-β5, anti-β6 and anti-β8 shows that αvβ6 and αvβ8 but not αvβ3 and αvβ5 are involved in TGFBR activation [86]. The same results are seen in human embryonic kidney (HEK) 293 T cells, where αvβ6 and αvβ8 are responsible for the release of active TGF-β, but αvβ1, αvβ3, and αvβ5 are not involved [87]. In contrast, αvβ8 but not αvβ6 mediates the release of active TGF-β in human fetal tracheal fibroblast cells [86]. Inhibiting TGF-β signals initiated by TGF-β1 and TGF-β3 in specific cells can be achieved by inhibiting integrins responsible for the activation of TGFBR while maintaining the ability of TGFBR to be activated by TGF-β2 and other mechanisms.

## Release of active TGF-β by reactive oxygen species

Asbestos is a well-reported carcinogen that causes lung injury by releasing cytokines, reactive oxidative species (ROS), and cell apoptosis [88, 89]. Mice exposed to asbestos have higher levels of Smad2 phosphorylation. When TEMPOL, an antioxidant, is administered to the mice before the introduction of asbestos, Smad2 phosphorylation is inhibited, demonstrating that asbestos-induced release of active TGF-β is mediated by ROS production [90]. In the presence of ascorbic acid, there is an increase in the release of active TGF-β in A549 lung cancer cells [91, 92]. Ascorbic acid-mediated release of active TGF-β is a unique mechanism for the liberation of active TGF-β1 but not other TGF-β isoforms [93]. LAP added to cell culture media containing active TGF-β suppresses the effect of TGF-β by inhibiting cell growth [94]. However, pre-incubating LAP with iron and ascorbic acid before adding to cell culture media containing TGF-β does not inhibit TGFBR activation, showing that ROS can modify the ability of LAP to maintain the latency of active TGF-β [91]. Further characterizing the sequence of LAP shows that methionine 253 is prone to iron and ascorbic-induced oxidation, enabling the release of active TGF-β [93]. Treating A549 lung cancer cells with nitric oxide (NO), a free radical, also stimulates the release of active TGF-β by disrupting the latency properties of LAP [95]. NO induction of TGFBR activation is cell-dependent as treatment with NO in bovine aortic endothelial cells prevents TGFBR activation and Smad2 nuclear translocation [96]. Asbestos, iron, ascorbic acid and NO produce ROS to oxidize susceptible residues in LAP, rendering LAP unable to maintain the latency of mature TGF-β in a cell-dependent manner.

Apocynin is a natural compound with nicotinamide adenine dinucleotide phosphate oxidase (NOX)- [97] and ROS-inhibiting properties [98, 99]. The antioxidative properties of apocynin are dependent on the cell type. In rat liver tissue [100] and macrophages [101], apocynin inhibited ROS production. However, in rat vascular fibroblasts [101], human vascular smooth muscle cells [102] and mouse microglial cells [103], apocynin stimulates the production of ROS. In the presence of myeloperoxidase, apocynin undergoes dimerization to form a more potent product [104]. Vascular cells such as rat aortic smooth muscle cells and porcine aortic endothelial cells do not express myeloperoxidase [105], which explains the effect of apocynin in enhancing ROS production seen in human vascular smooth muscle cells [102, 105].

In human vascular smooth muscle cells, apocynin-stimulated ROS production leads to the activation of TGFBR signaling by increasing Smad2 carboxyl-terminal phosphorylation [102]. Interestingly, apocynin does not inhibit NOX in human vascular smooth muscle cells, instead activating NOX to produce more intracellular ROS. Inhibiting NOX with a non-selective NOX inhibitor, diphenylene iodonium (DPI), suppresses ROS production and Smad2 carboxyl-terminal phosphorylation induced by apocynin, suggesting that TGFBR activation induced by apocynin is via NOX activation and ROS production [102]. Apocynin mediated Smad2 carboxyl-terminal phosphorylation is inhibited by ROCK inhibitor, Y2763, showing that apocynin activates TGFBR via the ROCK signaling pathway [102]. Activation of ROCK-dependent pathways controls the contraction of the cytoskeleton connected to RGD motif-recognizing integrins which facilitate the release of active TGF-β [102]. Intracellular ROS induced by apocynin stimulates the release of active TGF-β by employing the intracellular ROCK signaling pathway, whereas extracellular ROS-induced asbestos and NO directly modify the LAP structure to liberate active TGF-β.

While ROS induces TGF-β signaling, TGF-β also stimulates the production of ROS. In fetal rat hepatocytes, TGF-β upregulates the expression of NOX and the production of intracellular ROS, which is inhibited in the presence of DPI, showing that TGF-β and ROS form a vicious cycle [106]. A similar observation is seen in human pulmonary artery smooth muscle cells [107], human umbilical vein endothelial cells (HUVEC) [108] and rat kidney fibroblast [109]. Intracellular ROS can be targeted to regulate ROS levels and TGFBR activation simultaneously.

## Transactivation of TGFBR by different receptor signaling pathways

In addition to classical receptor signaling, receptors can communicate with each other to elicit downstream signaling responses, often termed receptor cross-talk or transactivation-dependent signaling [110]. Receptor transactivation is defined as the activation of the secondary receptor(s) triggered by the activation of a signaling pathway controlled by a primary receptor without the synthesis of a new agonist stimulating the secondary receptor(s) [30, 110]. The transactivated secondary receptor(s) is different from the primary receptor, modulating the cellular responses beyond the activation of the primary signaling pathway [30, 110]. A key parameter of transactivation dependent signaling is that it does not involve de novo synthesis of the secondary agonist. There are several examples where various cell surface receptors can transactivate TGFBR, leading to the downstream phosphorylation of Smads [111–113]. Receptor transactivation of the TGFBR involves the recruitment of RGD motif-recognizing integrins and/or MMPs to facilitate the release of active TGF-β in a short time without new TGF-β synthesis. The receptors demonstrated to transactivate TGFBR include multiple receptors of the G-protein coupled receptor (GPCR) families and toll-like receptor 4 (TLR4) [111, 114, 115].

The GPCR agonist, thrombin, signals via its cognate protease-activated receptor-1 (PAR-1) to transactivate TGFBR in mouse epithelial cells [116], fibroblasts [116], myofibroblast [117] and human vascular smooth muscle cells [111, 114, 118, 119]. In PAR-1-mediated TGFBR activation, the RGD motif-recognizing integrins but not MMPs control the Smad2 carboxyl-terminal phosphorylation [111, 114]. The RGD motif-recognizing integrins used by PAR-1 to activate TGFBR are found to be different in different cell types. αvβ6 is involved in mice lung epithelial cells [116] and fibroblasts [116], whereas αvβ5 but not αvβ1 and αvβ3 is involved in rat lung myofibroblast [117]. In human buccal mucosal fibroblast, αvβ1, αvβ3 and αvβ5 but not αvβ6 are used to mediate thrombin-stimulated release of active TGF-β, while αvβ5 and αvβ3 are equally utilized by human cardiac fibroblast [83]. Another ligand of PAR-1, FXa, via αvβ5-dependent pathways, transactivates the TGFBR in human lung fibroblast, leading to phosphorylation of Smad2 [120]. Inhibiting the RhoA/ROCK signaling pathway, which is upstream of cytoskeleton rearrangement, prevented PAR-1 transactivation of TGFBR [114, 116, 121], showing that PAR-1 via αvβ1, αvβ3, αvβ5 or αvβ6 and cytoskeleton rearrangement triggers the release of active TGF-β.

Lysophosphatidic acid (LPA) can signal via six respective LPARs (LPAR1–6) [122, 123] and different LPARs transactivate TGFBR. In mouse embryonic fibroblast [124], bronchial epithelial cells [124] and mouse proximal tubular cells [125], LPA activates LPAR2 but not LPAR1 and LPAR3 to recruit αvβ6 to activate TGFBRs, whereas LPAR5 but not LPAR1 and LPAR2 transactivates the TGFBR in human vascular smooth muscle cells [123]. αvβ5 is employed by LPA to stimulate TGFBR activation in human airway smooth muscle cells [126]. In normal human bronchial epithelial cells, LPA-induced TGFBR activation is inhibited by blebbistatin, an inhibitor of non-muscle myosin II which regulates the rearrangement of actin in the cytoskeleton [127]. Inhibiting the direct downstream effector of ROCK/RhoA activation—ezrin/radixin/moesin with curcumin, LPAR-stimulated TGFBR activation in human vascular smooth muscle is entirely suppressed [128]. Similar to the PAR-1 signaling pathway, LPAR activation employs ROCK/RhoA to trigger cytoskeleton rearrangement to modify the cellular architecture of integrins to activate TGFBR [124, 125, 128].

The GPCR agonist, endothelin-1 (ET-1), signals via ET-1 receptors (ET_A_ and ET_B_) to transactivate the TGFBR, leading to the phosphorylation of Smad2 carboxyl terminal in human vascular smooth muscle cells [129], bovine aortic endothelial cells [130, 131] and rat alveolar epithelial cells [132]. Different ET-1 receptors are involved in the transactivation of the two cell types. ET_A_ receptor transactivates TGFBR in rat alveolar epithelial cells [132], whereas ET_B_ is the responsible receptor in bovine aortic endothelial cells [130]. In bovine aortic endothelial cells, ET_A_ recruits ROCK to trigger cytoskeleton rearrangement to activate TGFBR [130]. However, in human vascular smooth muscle cells, ET-1 stimulates the activation of TGFBR via NOX, resembling the NOX/ROS-mediated activation of TGFBR observed with apocynin [133]. These studies show that ROCK or NOX signaling pathways are engaged by ET-1 to stimulate TGFBR activation in different cell types. However, more studies are warranted to differentiate if the selectivity of the mechanism in TGFBR activation is dependent on the subtype of ET-1 receptor as only ET_B_ initiates TGFBR activation, not ET_A_ in bovine aortic endothelial cells, which the recruitment of ROCK signaling pathway is observed [130].

In GPCR signaling pathways, different G proteins (Gαq, Gαi, Gαs and Gα12) convey the signals initiated by different agonists [134]. Mouse embryonic fibroblasts with silenced Gαq but not Gαi and Gα12/13 inhibited LPAR transactivation of the TGFBR [124]. Gαq inhibition with a pharmacological approach using G Protein antagonist-2A (GP-2A) in human bronchial epithelial cells [124] and molecular engineering approach using Gαq/11 minigene in mouse proximal tubular cells [125] show that LPAR-transactivation of the TGFBR is dependent on Gαq. Gαq is also used by PAR-1 to activate TGFBR. In human vascular smooth muscle cells, PAR-1 transactivation of TGFBR is inhibited by pan Gαq inhibitor, UBO-QIC, but not Gαq/11 inhibitor, YM254890, showing that PAR-1 signaling pathway recruits unique member(s) of Gαq family to transactivate TGFBR [119]. Family member(s) of Gαq might be universally responsible for the GPCR transactivation of TGFBR.

**Fig. 4:**
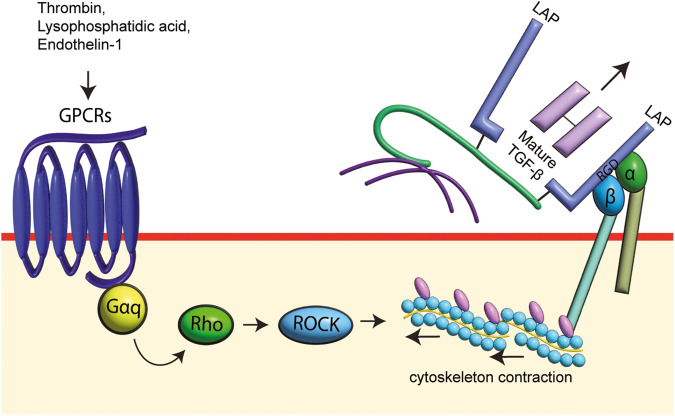
The GPCRs transactivation of TGFBR. Upon activation, G-protein coupled receptors (GPCRs) recruit Gαq to relay signals to Rho/ROCK, which will then activate cytoskeleton contraction to disrupt the structure of latency associated peptide (LAP) for the release of mature transforming growth factor (TGF)-β with the assistance of RGD motif-recognizing integrin. The active TGF-β will then bind to transforming growth factor-β receptor (TGFBR), fulfilling the command from GPCRs transactivation of TGFBR.

GPCR agonists, LPA, thrombin, and ET-1 via respective receptors transactivate the release of the active TGF-β. Mechanistic studies reveal that in most instances, GPCRs liberate the active TGF-β through interactions with RGD motif-recognizing integrins. Although no one specific integrin subunit was common to all the GPCRS, it appears that all GPCRs lead to the disruption of the cytoskeleton, a promoting conformational changes and leading to the release of active TGF-β (Fig. 4).

**Fig. 5:**
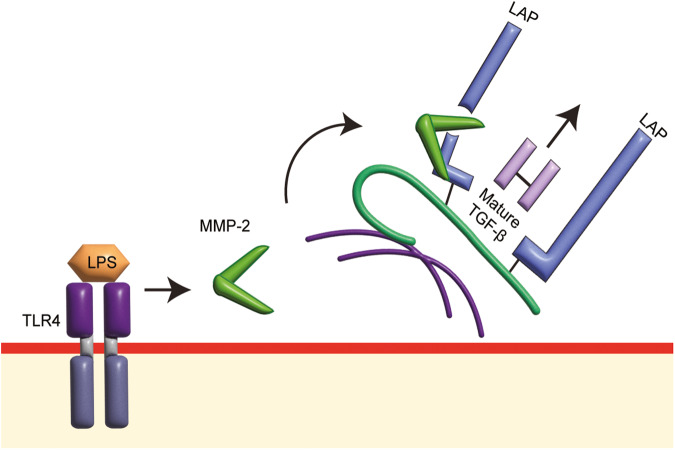
The TLR4 transactivation of TGFBR. Lipopolysaccharides (LPS)-bound toll-like receptor 4 (TLR4) activates matrix metalloproteinase-2 (MMP-2) but not matrix metalloproteinase (MMP)-9 to hydrolyze latency associated peptide (LAP). Hydrolyzed LAP loses the ability to maintain the latency of mature transforming growth factor (TGF)-β, allowing the release of mature TGF-β to activate transforming growth factor-β receptor (TGFBR), thereby relaying the signal from the TLR4 signaling pathway to the TGFBR signaling pathway.

In human vascular smooth muscle cells [115] and hepatic stellate cells [113], lipopolysaccharides (LPS) via its receptor, TLR4, induce TGFBR activation, leading to Smad2 carboxyl-terminal phosphorylation. In human vascular smooth muscle cells, TLR4-induced TGFBR activation involves MMPs but not ROCK-dependent pathways [115]. Employing pharmacological inhibitors of MMPs shows that TLR4-mediated TGFBR activation measured as phosphorylation of Smad2 carboxyl terminal in human vascular smooth muscle cells is dependent on MMP-2 but not MMP-9 dependent pathways [115]. Mitogen-activated protein kinases (MAPK) are common downstream mediators of receptor activation, including TGFBR, PAR-1 and TLR4 [135–138]. The recruitment of MAPK in the PAR-1 pathway does not lead to TGFBR activation and Smad2 carboxyl-terminal phosphorylation [135]. However, TLR4 transactivation of TGFBR is distinct from PAR-1. Members of the MAPK family have been shown to mediate TGFBR activation and Smad2 carboxyl-terminal phosphorylation. In hepatic stellate cells, p38 and Jnk inhibition abolish Smad2 carboxyl-terminal phosphorylation [113]. The same result is observed in human vascular smooth muscle cells, in which p38 is involved in TLR4-mediated TGFBR activation [115]. TLR4 transactivation of the TGFBR utilizes MAPK/MMP-dependent pathways, whereas GPCR transactivation of the TGFBR depends on ROCK/integrin-dependent pathways (Fig. 5). Inhibiting MAPK enables selective suppression of TGFBR activation initiated by TLR4 but not by GPCRs.

## Enhancing TGF-β signals by increasing the availability of TGFBRs

Upon TGFBR activation, TGFBR is internalized into clathrin/early endosome antigen (EEA)-1 endosome by the action of dynamin [25, 139]. Expression of dominant-negative dynamin K44E inhibits Smad2/3 translocation into the nucleus, showing that internalization of TGFBR into clathrin/EEA-1 endosomes is important for propagating Smad2/3 signaling [25]. Internalized TGFBRs reside in the cytoplasm and interact with AS160 and Rab11, which controls the reuse of TGFBRs to the plasma membrane for signal transduction [140, 141]. TGF-β signaling is initiated by TGFBR on the cell surface. Therefore, the abundance of TGFBR on the cell surface available for signaling transduction can regulate the sensitivity of a cell to TGF-β stimulation.

The cellular distribution of TGFBRs is asynchronous and cell-type specific. In many cells, a considerable portion of TGFBR resides in endosomes in cytoplasm, and only a small amount of TGFBR is found on the cell surface for signal transduction [140, 142, 143]. Intriguingly, agonists that activate the kinase, Akt, can lead to an increase in the abundance of TGFBRI and TGFBRII on the cell surface by phosphorylating AS160 [144]. TGF-β and high glucose (25 mM) induced Akt phosphorylation, enhances the availability of TGFBRs on the cell surface for signal transduction in HaCaT keratinocytes [143] and mouse epithelial fibroblasts [142], showing that TGF-β signals can be amplified with Akt activation. Insulin activates Akt in mouse epithelial fibroblasts [140] and HUVEC [144, 145], which then drives the translocation of TGFBR endosomes to the cell surface for signal transduction, leading to increased Smad2 phosphorylation. This process is quick as the increase in the abundance of TGFBR on the cell surface is observed as early as 15 min of exposure to insulin [140, 144]. However, without the release of active TGF-β from the TGF-β complex to activate TGFBRs, increasing the availability of TGFBR on the cell surface would add no value to TGF-β signaling. In mouse embryonic fibroblast [142] and rat kidney epithelial cells [142], high glucose (25 mM) increases the abundance of TGFBRs on the cell surface and the phosphorylation of Smad2/3. Inhibiting the MMPs with GM6001 in high glucose-treated cells drastically lowered the release of active TGF-β and the phosphorylation of Smad3, showing that the enhanced TGF-β signal requires the release of active TGF-β, which can bind to increased TGFBR on the cell surface to promote TGF-β signals [142]. Therefore, more studies are required to differentiate whether insulin-induced TGFBR activation and Smad2 phosphorylation involve the rapid release of active TGF-β.

SC79, an Akt activator, enables thrombin, the ligand of PAR-1, to activate TGFBRs in human keratinocytes, resulting in enhanced Smad2 carboxyl-terminal phosphorylation, which would be otherwise undetectable, even with prolonged exposure [146]. The exact mechanism is also observed in human vascular smooth muscle cells. Without Akt activation, angiotensin-II does not lead to observable TGFBR activation measured as Smad2 carboxyl-terminal phosphorylation. However, in the presence of SC79, TGFBR is activated, leading to a significant increase in Smad2 phosphorylation [146]. SC79 alone does not stimulate the phosphorylation of Smad2 in human vascular smooth muscle cells and keratinocytes. However, a time-dependent increase in Smad2 phosphorylation is observed when angiotensin-II or thrombin is added. These results demonstrate that the release of activated TGF-β by angiotensin-II or thrombin requires greater numbers of TGFBRs on the cell surface to elicit a measurable and potentially physiologically or pathophysiologically relevant response. Akt is a common mediator employed in the pathological states: epithermal growth factor receptor (EGFR) signaling pathway in non-small cell lung cancer [147], platelet-derived growth factor receptor signaling pathway in breast cancer cells [148] and vascular endothelial growth factor receptor signaling pathway in tumor angiogenesis [149]. Reviewing TGF-β signaling in diseases under the effect of Akt activation from other signaling pathways, will enable the discovery of suitable targets specific to disease-causing TGF-β signals while preserving the physiological requirement of cells on TGF-β signals. In addition, inhibiting TGF-β signaling by directly targeting TGFBR might be ineffective in disease states with enhanced Akt activity, in which cell responsiveness to TGF-β signaling is enhanced.

## Targeting TGFBR activation as a therapeutic approach

Therapeutics that target TGF-β signaling have predominately focused on inhibiting the TGFBR1 and TGF-β. Targeting the TGFBR inhibitors with biologicals and small chemical entities have been evaluated clinically for their effect in treating different types of cancers, and these include: vactosertib [150], galunisertib [151], LY3200882 [152] and LY3022859 (IMC-TR1) [153]. In addition, chemical entities SM16 [154, 155], SD208 [156] and LY2109761 [157] have demonstrated promising outcomes in inhibiting tumor cell growth in vivo and in vitro. Small molecule TGFBR inhibitors have also been investigated for use in fibrotic diseases. SM16 has anti-fibrotic effects in rats with vascular fibrosis in which the thickening of the blood vessel wall and lumen narrowing is reversed by SM16 [158]. In the mouse model of left ventricular fibrosis, SM16 administration attenuates Smad2 phosphorylation in the cardiac tissue and reduces collagen deposits in the left ventricle, associated with improved cardiac output [159]. In rats with Peyronie’s disease, Smad2 phosphorylation and the fibrotic area in the penile tissue are inhibited by vactosertib, ameliorating erectile dysfunction [160]. Vactosertib also inhibited TGF-β-induced fibronectin, collagen, and hydroxyproline synthesis in fibroblasts isolated from patients with Peyronie’s disease [160]. Several TGFBR inhibitors inhibit collagen and/or alpha-smooth muscle actin synthesis in different cells which include the use of galunisertib in human dermal fibroblast [161], LY2109761 in human hepatic stellate cells [162], hypertrophic scar fibroblast [163], and keloid-derived fibroblast [164] and SD208 in subsynovial connective tissue cells [165], CD14^+^ myocytes [166], and human primary dermal fibroblast [167]. These examples demonstrate the therapeutic potential of targeting the TGFBR to treat fibrosis.

Cardiotoxicity is a common side effect of anti-TGF-β therapy. Two small molecule inhibitors for TGFBR, AZ12601011 and AZ12799734, inhibit TGF-β induced human keratinocytes cell migration, and the former inhibits TGF-β induced tumor cell proliferation in vivo [168]. However, these drugs were not pursued clinically as they were associated with the formation of heart valve lesions in rats [169]. Cardiac valvulopathy is also observed in mice and cynomolgus monkeys treated with a pan-TGF-β antibody [170]. The assessment of cardiovascular effects of the TGFBR inhibitor, galunisertib, showed no cardiotoxic effects [171], however subsequent clinical trials with galunisertib excluded patients with moderate to severe cardiac disease [151, 172].

Targeting the interference between TGF-β and the TGFBR in the extracellular domain has also been exploited as a therapy. P144 [173], a protein derived from the TGF-β binding domain of betaglycan, works as a ‘TGF-β trap’, to inhibit Smad2 phosphorylation in the aorta and prevents the initiation of aneurysm in the murine model of Marfan syndrome. The therapeutic potential of P144 is also seen in rats with spontaneous hypertension, where treatment was associated with an increase in collagen in the myocardium [174] and the production of ROS in the glomeruli tissue [175]. Another similar example, RER [176], the hybrid protein of the extracellular domains of betaglycan and TGFBRII, inhibits the proliferation and invasiveness of prostate tumor cells in vitro and reduces the invasion of adenocarcinoma cells in mice with prostate cancer. The extracellular domain of TGFBR can be fused with therapeutic antibodies to form a new type of drug called bifunctional fusion protein or Y-trap [177]. In the tumor microenvironment, programmed death-ligand 1 (PD-L1) plays a role in suppressing immune cell activation and enables tumor cells to escape from immune surveillance [178]. PD-L1 antibody hybridized with the extracellular domain of TGFBRII promotes the anti-cancer effects of PD-L1, the inhibition of TGF-β by PD-L1 enhances the activation of immune cells to destroy tumor cells [177]. SHR-1701 [179] and bintrafusp alfa (M7824) [180] are formed by the fusion of PD-L1 antibody with the extracellular domain of TGFBRII. Bintrafusp alfa has been assessed for use in squamous cell carcinoma of the head and neck [180] and biliary tract cancer [181], but the clinical benefit of bintrafusp alfa is limited with increased bleeding risk, which may require careful titration of dose or treatment discontinuation [182]. Recently, a Y-trap drug prepared by hybridizing an EGFR antibody with the extracellular domain of TGFBRII, BCA101, has increased the synthesis of natural killer cells or T cells activating cytokines. BCA101 reduces the tumor volume more than the combination of TGFBRII extracellular domain protein and cetuximab in vivo [183].

In addition to targeting the TGF-β or TGFBRs, approaches such as targeting intermediates associated with TGFBR activation have also been utilized as therapeutics to target TGF-β signaling. αvβ6 targeting antibody, 264RAD [184], binds to the LAP domain of TGF-β and inhibits squamous carcinoma cell growth in vivo. Another antibody of αvβ6, BG00011 (STX-100), inhibits the binding of αvβ6 to latent TGF-β and was investigated in a phase IIb clinical trial for idiopathic pulmonary fibrosis [185]. However, the BG00011 clinical trial was terminated due to poor clinical benefit and is associated with increased death [185]. Abituzumab [186], a pan-αv antibody inhibits the release of active TGF-β in the Detroit 562 cancer cells and suppresses Detroit 562 cancer cell proliferation. TTB is a hybrid protein formed by fusing RGD peptide to the extracellular domain of TGFBRII [187]. TTB lowers the level of all TGF-β isoforms to a greater extent when compared to the pan-TGF-β antibody, 1D11 or TGF-β trap derived from the extracellular domain of betaglycan. In addition, TTB inhibits the proliferation and invasiveness of A549 lung cancer cells and 4T1 breast cancer cells more than the TGF-β trap [187]. The example of TTB demonstrates that the mechanisms of TGFBR activation can be explored as targeted therapies to enhance the effect of anti-TGF-β treatment.

## Conclusion and perspectives

In the TGFBR signaling pathway, TGFBR activation leads to the phosphorylation of Smad2/3, which will then associate with Smad4 and co-factors before transportation to the nucleus and modulation of target gene expression. The mechanisms of TGFBR activation are as versatile as the TGFBR signaling pathway, as numerous mediators are involved in liberating active TGF-β for the activation of TGFBR. Active TGF-β can be released by physical means, such as employing a cytoskeleton connected to RGD motif-recognizing integrin to disrupt the architecture of latent TGF-β or by using motifs on TSP-1, F-spondin and neuropilin-1 to unwind the complex structure of latent TGF-β for the release of active TGF-β. The release of active TGF-β can also be achieved by directly modifying the configuration of latent TGF-β with MMPs and reactive oxygen species. These mechanisms require specific cell compartments for action and are unique to cell types and TGF-β isoforms. This high level of control enables precise regulation of extracellular TGF-β signals, which is essential for managing TGFBR activation and its downstream comprehensive biological effects. This review exposes the plethora of approaches to inhibiting TGFBR activation and, thus, the untapped opportunities to modulate the role of TGF‑β and its downstream effectors, the Smad transcription factors, in the many pathological situations in which they have been implicated.
